# Prevalence, characteristic features, and complications associated with the occurrence of unerupted permanent incisors

**DOI:** 10.1371/journal.pone.0199501

**Published:** 2018-06-28

**Authors:** Chiewee Tan, Manikandan Ekambaram, Cynthia Kar Yung Yiu

**Affiliations:** Paediatric Dentistry, Faculty of Dentistry, The University of Hong Kong, Pokfulam, Hong Kong SAR, China; Ohio State University, UNITED STATES

## Abstract

This study examined the prevalence, characteristic features, and complications associated with the occurrence of unerupted permanent incisors among children and adolescents attending a university dental teaching hospital. A retrospective review was performed of the clinical records of children and adolescents who attended the Prince Philip Dental Hospital, Hong Kong between 2005 and 2014. All patients who had at least one unerupted permanent incisor tooth were included. A total of 266 subjects with 320 unerupted permanent incisors were identified. The prevalence of unerupted permanent incisors among children and adolescents was 2.0%. Permanent maxillary central incisors (70.6%) were the most commonly affected teeth. The most common cause for unerupted incisors were dilacerations (n = 83, 36.7%) for maxillary central incisors; developmental dental anomalies (n = 22; 30.6%) together with unfavorable root development (n = 22; 30.6%) for maxillary laterals incisors; and abnormal tooth/tissue ratio (n = 11, 50.0%) for mandibular incisors. A majority of unerupted incisors presented with complications the most common being ectopic/displacement/rotation of the unerupted incisors (46.6%), loss of space (36.9%) and midline shift (27.5%). In conclusion, the causes were distinct for different manifestations of unerupted permanent incisors. As the majority of unerupted incisors presented with complications, a systematic and organized method of history taking as well as clinical and radiographic examinations is mandatory in the diagnosis of unerupted permanent incisors.

## Introduction

Delayed eruption of maxillary permanent incisors necessitates intervention when [1]

1. there is eruption of contralateral teeth that occurred greater than six months previously;
2. the lower incisors have erupted greater than one year previously and where both maxillary central incisors remain unerupted;
3. or there is deviation from the normal order of eruption (e.g. lateral incisors erupting prior to the central incisors).

A previous study reported that the incidence of unerupted maxillary incisors in the 5 to 12 year-old age group as 0.13% [2] and more prevalent in Asian races [1]. The etiological factors for delayed eruption of maxillary incisors can be classified into two main categories, namely hereditary and environmental factors. Hereditary factors include supernumerary teeth, cleft lip and palate, odontoma, abnormal tooth/tissue ratio, cleidocranial dysostosis, generalized delayed eruption and gingival fibromatosis. Similarly, trauma, early extraction or loss of primary teeth (with or without space loss), retained primary teeth, cystic formation, endocrine abnormalities, and bone disease are environmental factors that may influence eruption of incisors [1].

To date, published studies have focused on case reports of diagnosis and management of unerupted maxillary teeth [3], retrospective evaluation on eruption [4]; treatment protocol [5]; prevalence of various etiological factors and treatment outcome associated with unerupted incisors [6]; as well as association between unerupted incisors and dental anomalies [7]. However, there is no single study on prevalence, characteristic features and complications associated with unerupted permanent incisors among children and adolescents attending the Prince Philip Dental Hospital (PPDH), which is the only university dental teaching hospital in Hong Kong Special Administrative Region, China. The patient pool in Pediatric Dentistry and Orthodontics (PDO) clinic of PPDH are predominantly those without a referral, followed by referred patients from the School Dental Care Services (a dental health program for almost all primary school children); private general dental practitioners and medical practitioners. Almost all children and adolescents with unerupted permanent incisors are accepted for comprehensive multidisciplinary treatment in the PDO clinic. Therefore, the aim of this retrospective study was to determine the prevalence, characteristic features and complications associated with the occurrence of unerupted permanent incisors among children and adolescents attending a university dental teaching hospital.

## Materials and methods

This retrospective study reviewed hospital records of children and adolescents who had unerupted permanent incisors and were treated in the PDO clinic at the PPDH, The University of Hong Kong, Hong Kong SAR, between January 2005 and December 2014. The study protocol was approved by the Institutional Review Board of the University of Hong Kong/Hospital Authority Hong Kong West Cluster (UW 15–538). The records were reviewed from 15,987 children and adolescents who had attended the PDO Clinic of the PPDH. All patients who had at least one unerupted permanent incisor that matched the definition of unerupted permanent incisors which was adapted from the clinical guidelines of Royal College of Surgeons of England [1] were included. Those subjects were defined as having unerupted permanent incisors when any of the following occurred:

- eruption of contralateral incisors that occurred greater than six months previously;
- both maxillary central incisors remain unerupted and the lower incisors have erupted greater than one year previously;
- there is deviation from the normal sequence of eruption (e.g. lateral incisors erupting prior to the central incisors).

Clinical records of those patients were excluded from this study if they presented with

- medical complications (metabolic and endocrine disorders, syndromes, orofacial cleft / craniofacial malformations etc.)
- congenital missing permanent incisors.

After obtaining a list of included patients, clinical notes and radiographs were retrieved to obtain the necessary information. A customized data entry form was used to record all relevant information of each patient. Two qualified dentists who worked in the PDO clinic of the PPDH were involved in the data collection stage. The assessment process of clinical reports and radiographs was blinded. The inter- and intra-examiner reliability were assessed by using the Cohen’s Kappa statistics.

For each patient included in the study, the relevant information that was recorded included:

1. General descriptive data about the patients that includes gender, date of birth, date of diagnosis
2. Relevant clinical information such as,
  - The type of radiographs used for assessment and diagnosis of unerupted incisors
  - Type, number and etiology of unerupted incisors
  - History of trauma
  - The types of supernumerary and odontomas associated with unerupted incisors
  - Any complications associated with the occurrence of unerupted permanent incisors

All the data were entered and processed using the Statistical Package for Social Sciences Version 20.0 for Windows® (SPSS Inc., Chicago, Illinois, USA). Numerical data were summarized. Any significant differences and possible associations among variables were compared using Chi-square test. Fisher’s exact test was used when 20% or more of the cells in the table with expected frequencies less than 5. The binomial test was used to determine any difference in between proportion of presence and absence of complications associated with the occurrence of unerupted permanent incisors. The p-value was set at 0.05.

## Results

### General descriptive data

A total of 266 subjects with 320 unerupted permanent incisors were identified from the 15,987 subjects in the study, which represented a prevalence of 2.0%. Out of 266 subjects, male subjects (n = 139; 52.3%) were slightly higher than female (n = 127; 47.7%). The age of diagnosis ranged from 7.3 years to 13.8 years with a mean age of 10.6 years (S1 Table).

### Type of radiographs used

There were good intra-examiner and inter-examiner reliability with Kappa values ranging from 0.85 to 0.87 and 0.80 to 0.83, respectively. Panoramic (OPG), upper anterior occlusal view (UAO) and periapical (PA) view radiographs were the most common combinations of radiographs used for assessment and diagnosis of unerupted incisors (n = 115, 35.9%). Only 46 cases (14.4%) incorporated Cone Beam Computed Tomography (CBCT) in diagnosis and treatment planning of unerupted incisors.

### Type and number of unerupted incisors

**Fig 1:**
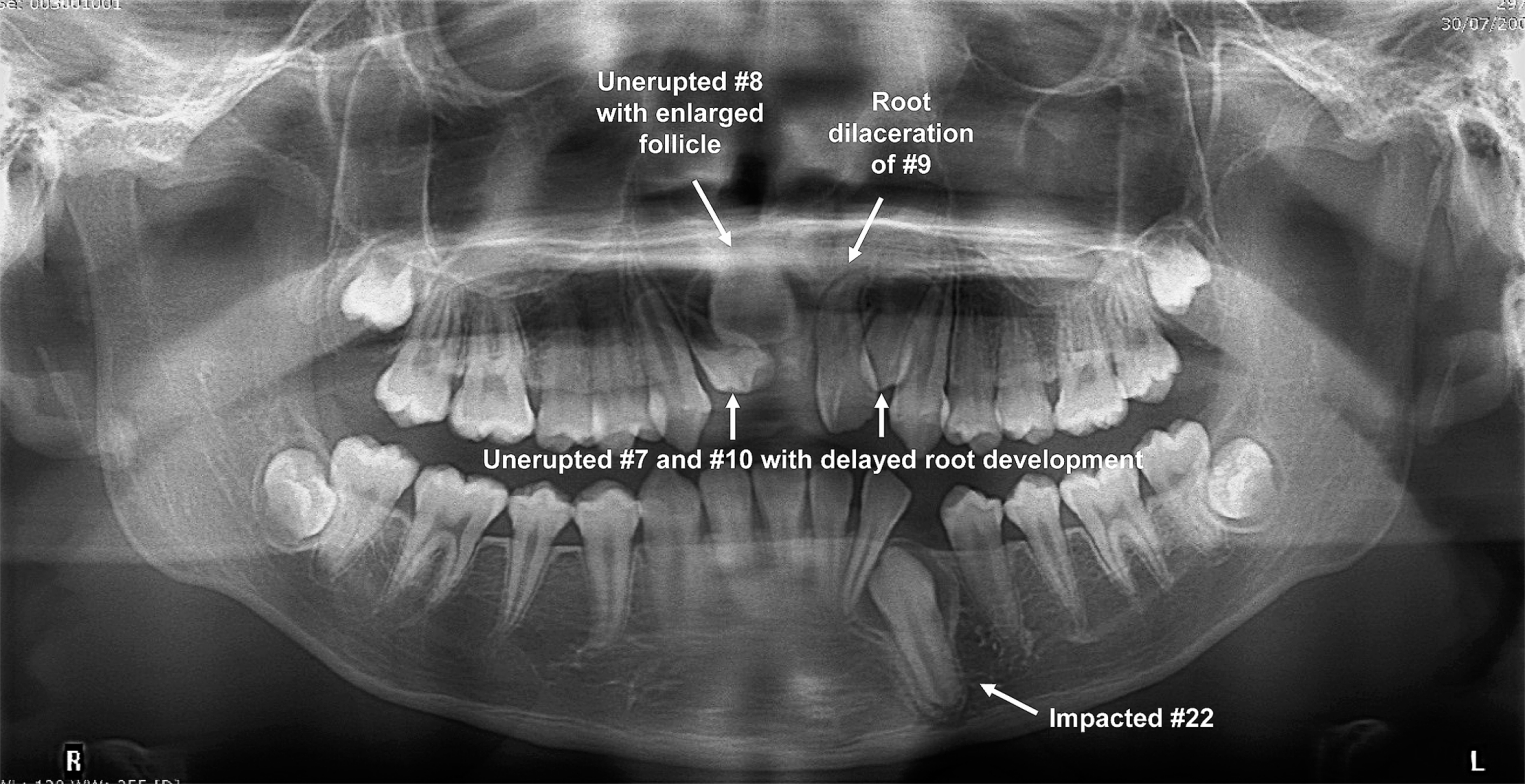
Panoramic radiograph of a 14-year-old girl (with no history of dental trauma) presented with root dilaceration of #9, unerupted #8 with enlarged follicle, unerupted #7 and #10 with delayed root development; as well as an impacted #22.

Among the unerupted permanent incisors, maxillary central incisors were the most frequently affected teeth (70.6%), followed by maxillary lateral incisors (22.5%), mandibular lateral incisors (4.1%) and mandibular central incisors (2.8%) (Table 1). Most of the patients presented with only one unerupted permanent incisor (n = 220; 68.8%) and 86 patients (26.9%) had two unerupted permanent incisors involved. There were only 6 (1.9%) (as shown in Fig 1) and 8 (2.5%) patients that presented with three and four unerupted permanent incisors, respectively.

**Table 1:** Prevalence and percentages of different types of unerupted permanent incisors.

Types of incisors involved	Prevalence	Tooth number	n (%)
Maxillary centrals (n = 226; 70.6%)	1.41%	#8	104 (32.5)
		#9	122 (38.1)
Maxillary laterals (n = 72; 22.5%)	0.45%	#7	41 (12.8)
		#10	31 (9.7)
Mandibular centrals (n = 9; 2.8%)	0.06%	#24	7 (2.2)
		#25	2 (0.6)
Mandibular laterals (n = 13; 4.1%)	0.08%	#23	5 (1.6)
		#26	8 (2.5)
Total	2.00%		320 (100.0)

### Etiology of unerupted incisors

**Fig 2:**
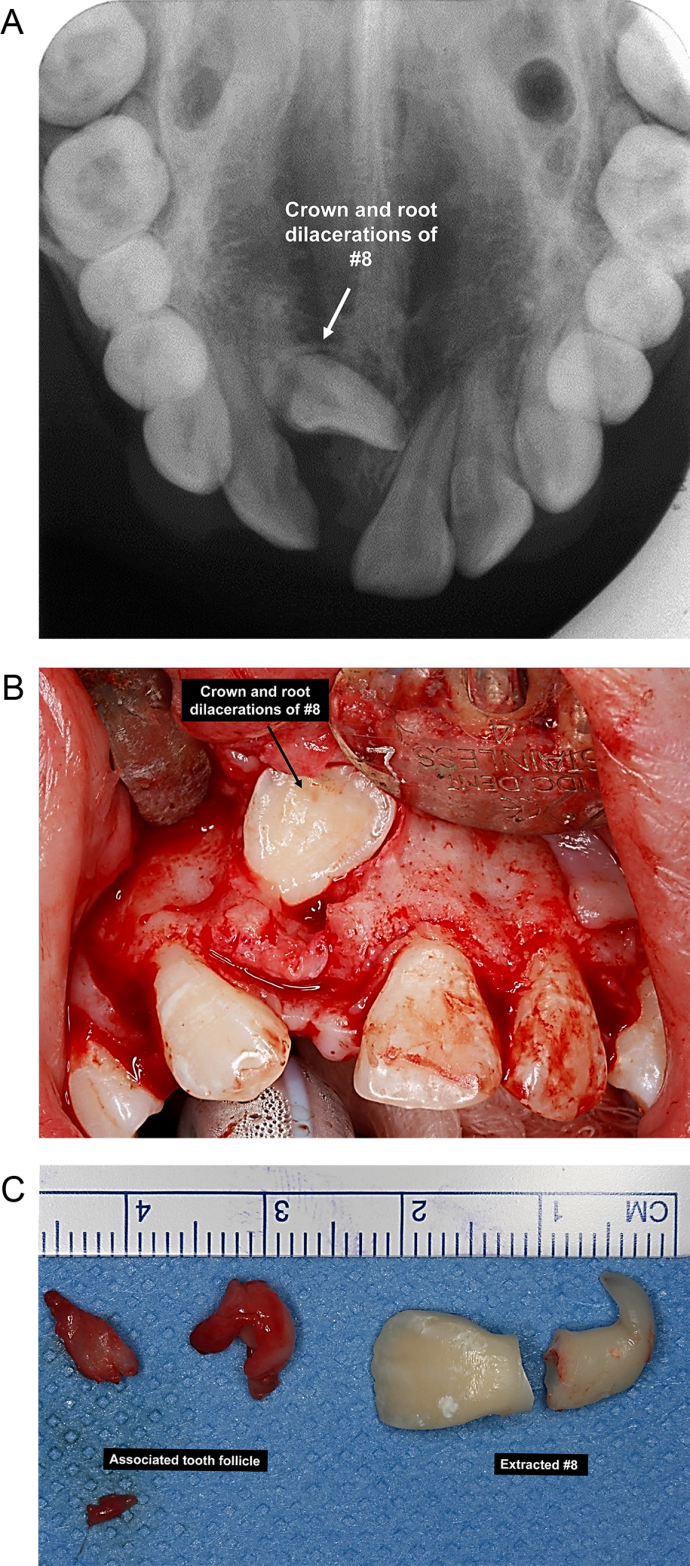
a. Upper anterior occlusal view of a 12-year-old girl who presented with a crown and root dilacerations of #8 as well as positive history of traumatic injury to the primary incisor when she was 4-year-old. b. Intra-operative photograph of the same subject which shows the orientation of the dilacerated crown. c. Postoperative photograph of the same subject with the extracted #8 and its associated tooth follicle.

**Fig 3:**
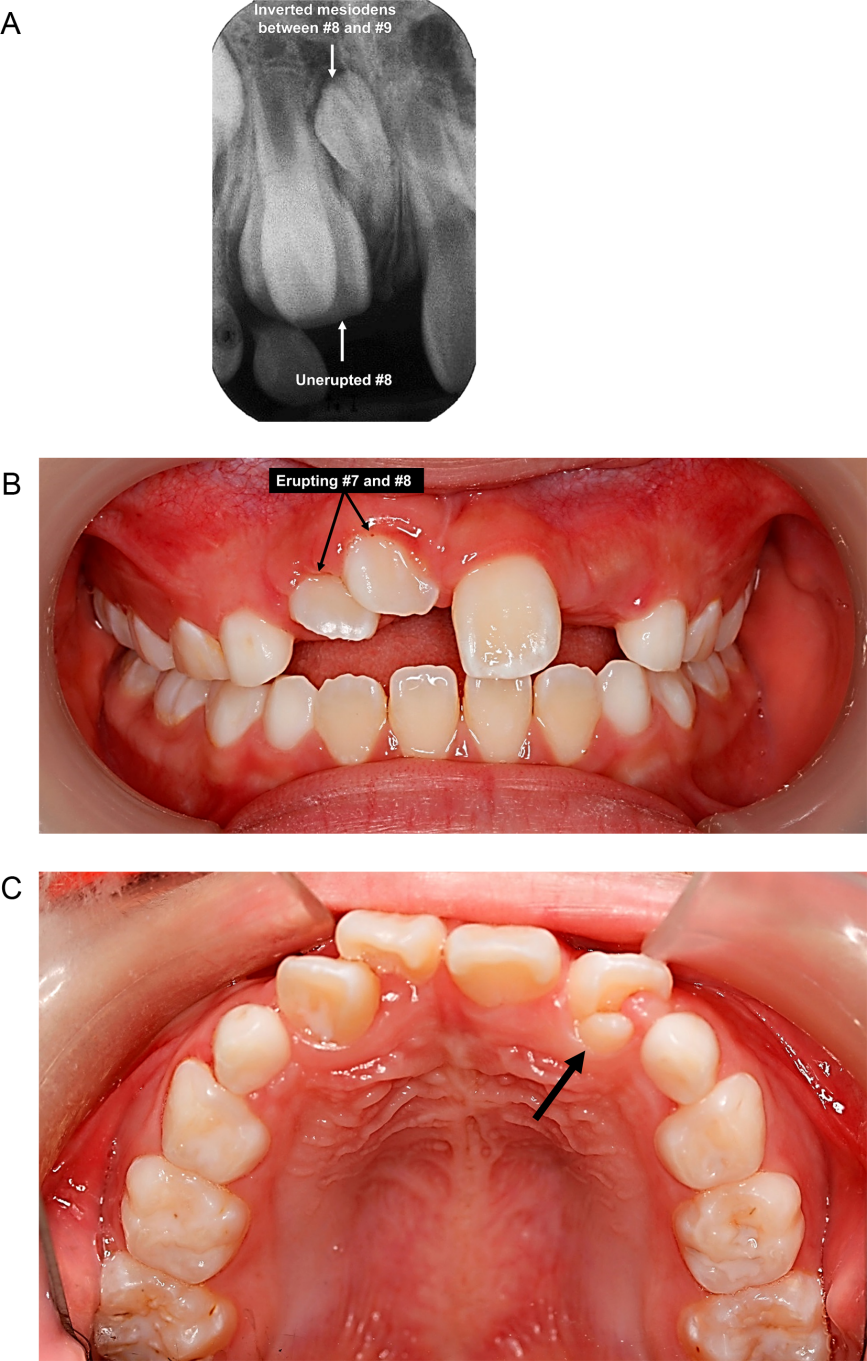
a. Periapical radiograph of an 8-year-old boy who presented with an unerupted #8 due to the inverted mesiodens between #8 and #9. b. Frontal view of the same subject with erupting #7 and #8 after one month of surgical removal of the inverted mesiodens between #8 and #9. c. Occlusal view of the same subject showing talon’s cusps and dens invaginatus on palatal surface of erupted #10 (black arrow) after six months of surgical removal of the inverted mesiodens between #8 and #9.

There were in total 14 causes of unerupted permanent incisors that were identified and categorized under either “hereditary factors” or “environmental factors” (Table 2). There were five cases (1.6%) with unknown/unspecified etiology. Overall, the three most common causes for unerupted permanent incisors were dilacerations (n = 88, 27.5%), followed by supernumerary teeth (n = 66, 19.1%) and ectopic position of tooth bud (n = 52, 16.3%). For the unerupted maxillary central incisors, the most common causes were dilacerations (n = 83, 36.7%) (Fig 2A–2C), followed by supernumerary teeth (Fig 3A–3C) (n = 49; 21.7%) and ectopic position of tooth bud (n = 32; 14.2%). For the unerupted maxillary lateral incisors, the three most common causes were delayed root development (n = 21; 29.2%); malformed/microdontic/underdeveloped incisors (n = 20; 27.8%) and ectopic position of tooth bud (n = 15; 20.8%). The most common cause of unerupted mandibular incisors was crowding (abnormal tooth/tissue ratio), which included 5 cases (38.5%) for mandibular lateral incisors and 6 cases (66.7%) for mandibular central incisors. Many of the cases presented with one underlying etiology of unerupted permanent incisors (n = 243; 75.9%); whereas 64 cases (20%) and 7 cases (2.2%) presented with a combination of two and three causes. Only one case (0.7%) presented with combination of 4 underlying causes which includes compound odontomas and root dilaceration in relation to #9, as well as retained #F and delayed root development of #9 compared with #8.

**Table 2:** Identified causes of unerupted permanent incisors.

		Overall n (%)	Maxillary centrals n (%)	Maxillary laterals n (%)	Mandibular centrals n (%)	Mandibular laterals n (%)
**A.**	**Hereditary causes**					
1.	Supernumerary teeth	61 (19.1)	49 (21.7)	11 (15.3)	0 (0.0)	1 (7.7)
2.	Abnormal tooth/tissue ratio (crowding)	29 (9.1)	7 (3.1)	11 (15.3)	6 (66.7)	5 (38.5)
3.	Developmental dental anomalies	29 (9.1)	6 (2.6)	22 (30.6)	0 (0.0)	1 (7.7)
a.	Malformed/microdontic/ underdeveloped	23 (7.2)	3 (1.3)	20 (27.8)	0 (0.0)	0 (0.0)
b.	Regional odontodysplasia	6 (1.9)	3 (1.3)	2 (2.8)	0 (0.0)	1 (7.7)
4.	Odontomas	21 (6.6)	19 (8.4)	1 (1.4)	1 (11.1)	0 (0.0)
5.	Generalized delayed eruption	4 (1.3)	2 (0.9)	2 (2.8)	0 (0.0)	0 (0.0)
**B.**	**Environment causes**					
6.	Dilacerations	88 (27.5)	83 (36.7)	5 (7.0)	0 (0.0)	0 (0.0)
a.	Root dilacerations	33 (10.3)	30 (13.3)	3 (4.2)	0 (0.0)	0 (0.0)
b.	Crown dilacerations	31 (9.7)	29 (12.8)	2 (2.8)	0 (0.0)	0 (0.0)
c.	Crown and root dilacerations	24 (7.5)	24 (10.6)	0 (0.0)	0 (0.0)	0 (0.0)
7.	Ectopic position of tooth bud	52 (16.3)	32 (14.2)	15(20.8)	2 (22.2)	3 (23.1)
8.	Retained primary teeth	38 (11.9)	23 (10.2)	10 (13.9)	1 (11.1)	4 (30.8)
9.	Unfavorable root development	37 (11.6)	14 (6.2)	22 (30.6)	0 (0.0)	1 (7.7)
a.	Delayed root development	31 (9.7)	9 (4.0)	21 (29.2)	0 (0.0)	1 (7.7)
b.	Arrested root development	6 (1.9)	5 (2.2)	1 (1.4)	0 (0.0)	0 (0.0)
10.	Obstruction by adjacent teeth	15 (4.7)	5 (2.2)	10 (13.9)	0 (0.0)	0 (0.0)
11.	Early extraction or loss of primary teeth	14 (4.4)	14 (6.2)	0 (0.0)	0 (0.0)	0 (0.0)
a.	With space loss	0 (0.0)	0 (0.0)	0 (0.0)	0 (0.0)	0 (0.0)
b.	Without space loss	14 (4.4)	14 (6.2)	0 (0.0)	0 (0.0)	0 (0.0)
12.	Pathological changes—Cystic formation etc.	5 (1.6)	5 (2.2)	0 (0.0)	0 (0.0)	0 (0.0)
13.	Ankyloses/ impaction of primary teeth	2 (0.6)	0 (0.0)	2 (2.8)	0 (0.0)	0 (0.0)
14.	Ankyloses of permanent teeth	1 (0.3)	0 (0.0)	0 (0.0)	1 (11.1)	0 (0.0)

### History of dental/ maxillofacial trauma

Among all the 14 causes of unerupted permanent incisors, dilacerations (p<0.001), ectopic position of tooth bud (p = 0.012) and ankylosed/impacted primary teeth (p = 0.039) had significant association with positive history of trauma.

### Types of supernumerary and odontomas associated with unerupted incisors

Among the type of supernumerary teeth associated with unerupted permanent incisors, the conical type of supernumerary tooth (n = 34, 55.7%) was significantly higher in number when compared with tuberculate (n = 12, 19.7%), supplemental supernumerary teeth (n = 10, 16.4%) and unspecified (n = 5, 8.2%) types (p<0.001). Conversely, compound odontomas (n = 17, 81.0%) had a significant higher proportion when compared with complex odontomas (n = 0, 0.0%) (p<0.001).

### Associated complications with the occurrence of unerupted incisors

Overall, a significantly higher number of unerupted incisors presented with associated complications (n = 243; 75.9%) than those without complications (n = 77; 24.1%) (p<0.001). The complications associated with the unerupted permanent incisors can be categorized under general dental aspect, associated with unerupted incisors, associated with adjacent teeth/structures (Table 3). The unerupted maxillary central incisors were found to be most frequently associated with ectopic/displacement/rotation of the unerupted permanent incisor itself (n = 111; 49.1%), space loss (n = 93; 41.2%) and midline shift (n = 61; 27.0%). For the unerupted maxillary lateral incisors, ectopic/ displacement of the unerupted incisor itself (n = 30; 41.7%) and its adjacent teeth/structures (n = 19; 26.4%) were the two most common complications associated with the occurrence of unerupted permanent incisors. A majority of unerupted mandibular permanent incisors presented with space loss (n = 8; 88.9%) and midline shift (n = 8; 88.9%).

**Table 3:** Complications associated with the occurrence of unerupted permanent incisors.

		Overall n (%)	Maxillary centrals n (%)	Maxillary laterals n (%)	Mandibular centrals n (%)	Mandibular laterals n (%)
**1.**	**General dental aspects**					
a.	Loss of space	118 (36.90)	93 (41.2)	12 (16.7)	8 (88.9)	8 (88.9)
b.	Midline shift	88 (27.50)	61 (27.0)	12 (16.7)	8 (88.9)	8 (88.9)
**2.**	**Associated with unerupted incisors**					
a.	Ectopic/displacement/rotation	149 (46.60)	111 (49.1)	30 (41.7)	2 (22.2)	2 (22.2)
b.	Enlarged follicle	53 (16.6)	37 (16.4)	10 (13.9)	5 (55.6)	1 (7.7)
c.	Cystic changes	5 (1.60)	5 (2.2)	0 (0.0)	0 (0.0)	0 (0.0)
**3.**	**Associated with adjacent teeth/structures**					
a.	Ectopic/displacement/rotation	83 (25.90)	58 (25.7)	19 (26.4)	1 (11.1)	1 (11.1)
b.	Enlarged follicle	3 (0.9)	2 (0.9)	0 (0.0)	1 (11.1)	0 (0.0)
c.	Root resorption	5 (1.60)	0 (0.0)	2 (2.8)	0 (0.0)	0 (0.0)
d.	Early exfoliation	4 (1.30)	0 (0.0)	1 (1.4)	0 (0.0)	0 (0.0)
e.	Overeruption of opposing incisors	8 (2.50)	7 (3.1)	1 (1.4)	0 (0.0)	0 (0.0)
**4.**	**No complication**	77 (24.10)	57 (25.2)	18 (25.0)	0 (0.0)	0 (0.0)

## Discussion

The demographic data in our study showed that male subjects (n = 139; 52.3%) were slightly higher than female (n = 127; 47.7%) with a mean age of 10.6 years. This is in accordance with other retrospective studies, which also reported higher prevalence of unerupted maxillary central incisors in males with a mean age of 10.6 and 9.4 years [5, 8]. The contributing factors for unerupted incisors among male patients could be explained by a greater prevalence of supernumerary teeth in males [9, 10] and also possible involvement of sexual chromosomes in the etiology of tooth eruption disturbances [8].

In the present study, the most common combination of radiographs that were used in assessment and management of unerupted permanent incisors were panoramic radiograph, UAO view and PA view radiographs. This is like another retrospective study [10], in which a combination of more than one film was used in 95% of cases to enable localization of the unerupted teeth using the parallax method. A study by Jacobs [11] suggested that a panoramic radiograph with an anterior occlusal radiograph is the preferred combination of radiographs to localize unerupted mandibular anterior teeth. Radiographs may be justified when there are abnormal incisor relationships, localization of tooth position is necessary to formulate a treatment plan, and where a reasonable expectation that pathology exists on clinical grounds [12]. Cone Beam Computed Tomography (CBCT) could be used to provide valuable information in qualitative analysis of dento-osseous structures, morphological alterations and exact tri-dimensional positioning of the unerupted teeth and adjacent structures [13]. As a general principle, the recommendations from clinical guidelines of the Royal College of Surgeons of England [1] and the American Academy of Pediatric Dentistry [14] should be adopted during radiographic assessment of unerupted permanent incisors.

Findings from the present study showed diversities in the hereditary and environmental causes for each specific type of unerupted permanent incisors (Table 2). Trauma in the primary dentition has the potential to cause eruption disturbances of permanent incisors, especially maxillary central incisors as shown by this study. Acute trauma to the primary dentition can cause dilaceration of the long axis of the permanent successor [15]. Any trauma during odontogenesis can affect the morphogenic stages of dental development and malformations, such as partial or complete arrest of root formation which occur during root formation [16]. Development of odontoma-like malformations in the permanent dentition caused by intrusion of a primary incisor has also been reported [16, 17]. Therefore, the clinician should advise the parents regarding the possible complications following traumatic dental injuries of the primary dentition. Regular follow-up as recommended by the International Association of Dental Traumatology (IADT) guidelines [18] is also mandatory so that early detection and treatment of possible severe developmental disturbances can be carried out.

**Fig 4:**
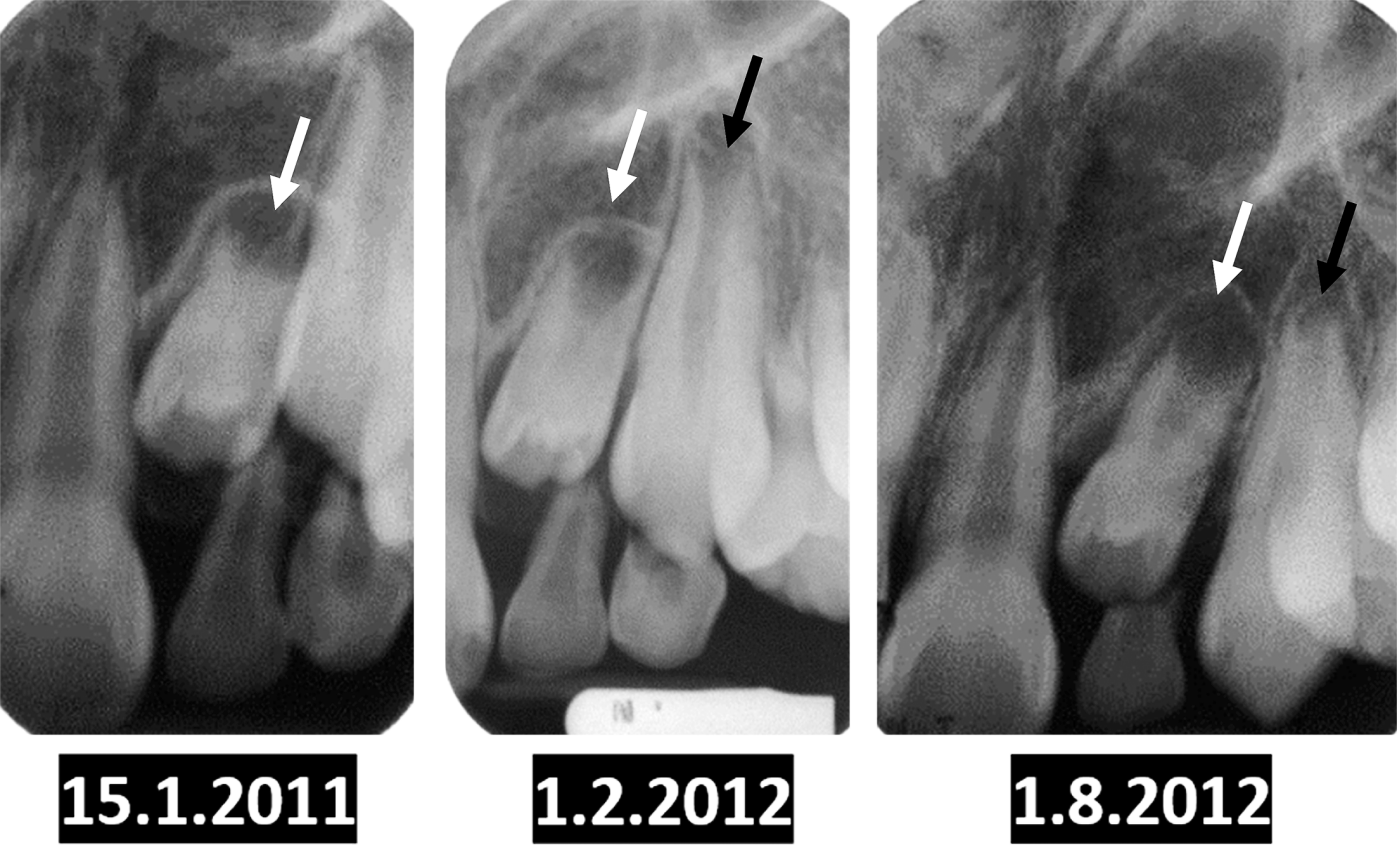
A 9 years old male subject who initially presented on 15^th^ January 2011, with malformed and delayed root development on #10 (white arrow). #11 (black arrow) had erupted in advance as compared to #10 on a review dated on 1^st^ August 2012. #10 had finally erupted clinically on the subsequent review dated on 21^st^ November 2012 (after 22 months of observation).

In addition, the results of this study also identified that the etiologies of unerupted incisors may also occur in isolation or in combination, specifically for maxillary permanent lateral incisors, which were most commonly affected by unfavorable root development (extremely delayed/arrested root development) and developmental dental anomalies (malformed/microdontic/underdeveloped) (Fig 4) [19, 20]. Surgical removal should only be performed when proven complications, such as obstruction of eruption, displacement or root resorption of adjacent teeth, or pathological cystic changes have occurred.

**Fig 5:**
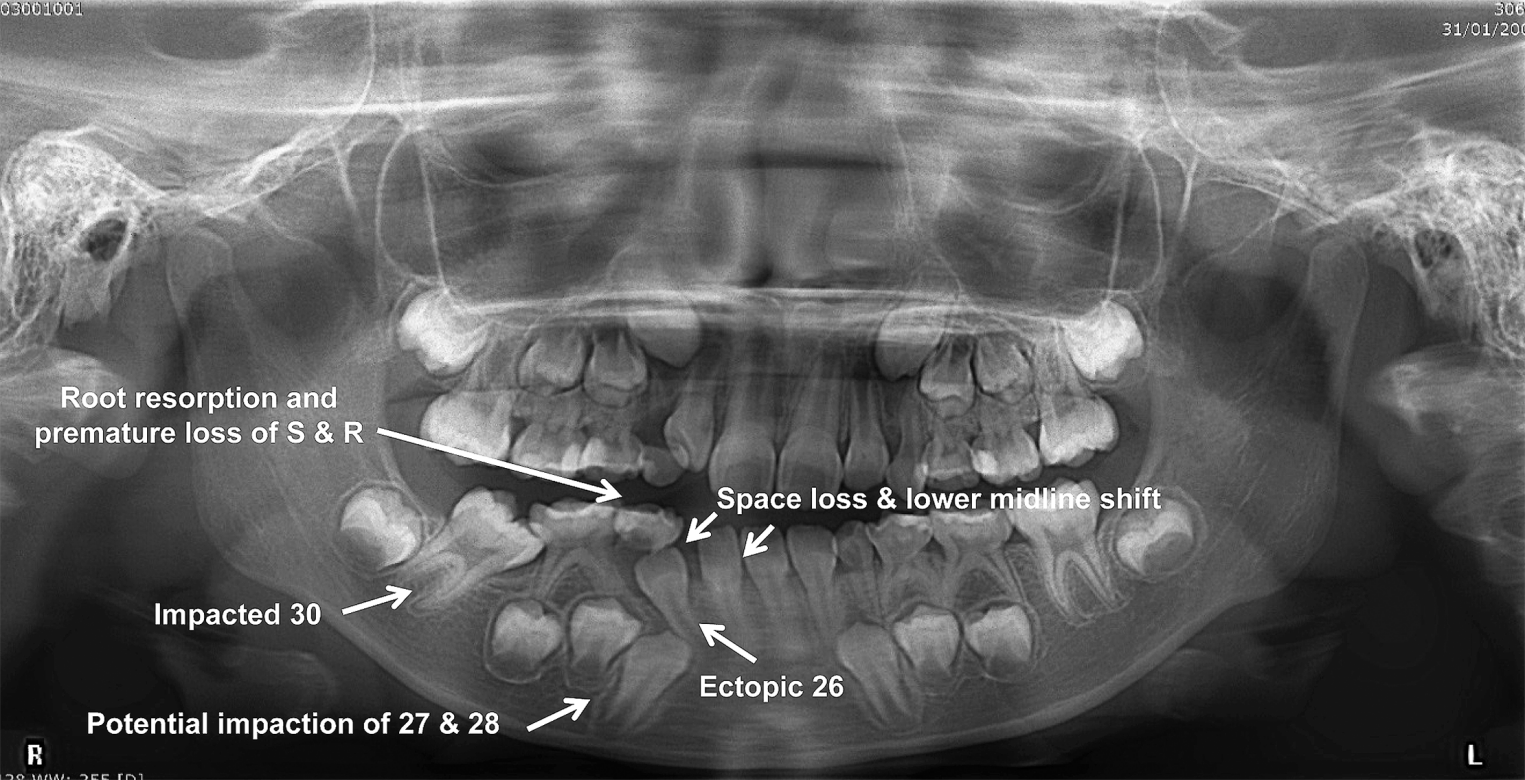
Panoramic radiograph of an 8-year-old boy showing the complications associated with the occurrence of an unerupted and ectopic #26.

Along with maxillary incisors, this study also addressed the occurrence and key characteristic features of 22 cases of unerupted mandibular incisors which were rarely reported in the literature likely due to its low prevalence. This study showed that causes for most unerupted mandibular incisors were due to crowding (abnormal tooth tissue ratio), followed by retained primary incisors and ectopic position of tooth buds. This condition was also often complicated with further space loss and midline shift as shown in Fig 5. Therefore, recognition of potential crowding during primary dentition and early mixed dentition by the clinician is the first important step in reducing the occurrence of unerupted mandibular incisors. Likewise, lack of space in the primary dentition during routine dental examination may raise the concern of future crowding in the permanent dentition [21]. Moreover, any over-retained primary tooth should be extracted to prevent obstruction to the eruption of the permanent successor [1].

**Fig 6:**
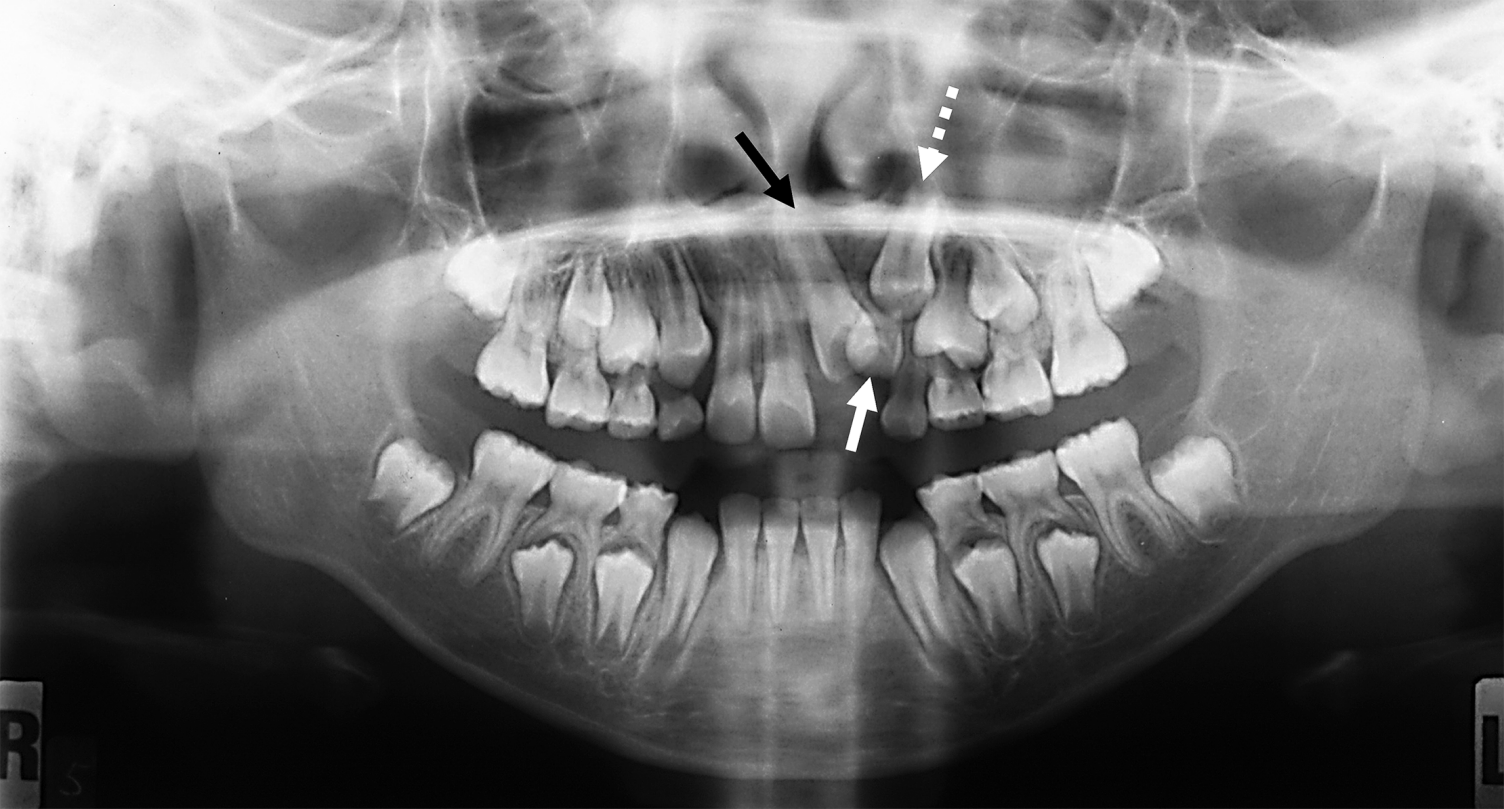
Panoramic radiograph of an 8-year-old boy showing the unerupted #10 with delayed root development (white arrow), which also acted as an obstruction to the eruption of #9 (black arrow) and #11 (dotted arrow).

Findings of the present study revealed that complications associated with the occurrence of unerupted permanent incisors can have an effect on the rest of the dentition (Fig 5), such as loss of space [22, 23] and midline shift [24, 25]. Other reported complications, which were also well documented in several published studies, include enlarged follicle or cystic changes of unerupted incisors [26, 27] or association with supernumerary teeth [28, 29], root resorption and early exfoliation of adjacent teeth. Complications may also be associated with ectopic/ displacement/ rotation of the impacted incisor itself [30, 31] or the adjacent structures/ teeth [32]. For instance, a microdont maxillary lateral incisor displaying delayed development may inhibit eruption of maxillary central incisor(s) and maxillary canine(s) (Fig 6). In a case series, Kobayashi et al. [19] reported that developmental anomalies and immaturity involving adjacent permanent lateral incisors could be associated with eruption failure of maxillary permanent central incisors. However, the immature tooth germ does not change the eruptive direction of the tooth as much as do the presence of odontomas and supernumerary teeth, even if the developing tooth germ is positioned close to the unerupted tooth [19]. Nevertheless, early detection of unerupted permanent incisors would be essential to prevent the impact of the above-mentioned complications, reduce the treatment complexity and thus improve the clinical outcome.

Due to the retrospective nature of the present study, the major limitation was information bias which could have led to missing data for analysis and interpretation. This was also compounded by the lack of completeness and standardized method of history taking, clinical and radiographic examinations in the information gathering process.

## Conclusions

1. The prevalence of unerupted permanent incisors among children and adolescents attending a university dental teaching hospital was 2.0%.
2. The most common etiologies for unerupted incisors were dilacerations for maxillary central incisors; developmental dental anomalies together with unfavorable root development for maxillary laterals incisors; and abnormal tooth/tissue ratio for mandibular incisors.
3. The majority of unerupted incisors presented with complications, which can have an effect on the remaining dentition (i.e. space loss and midline shift), as well as on the impacted incisor itself and/or its adjacent structures/teeth.

## Supporting information

S1 Table Original data of 266 subjects with unerupted permanent incisors from clinical record and radiographs. (XLSX)

## References

[1] Yaqoob O, O’Neill J, Patel S, Seehra J, Bryant C, Noar J, et al. Management of unerupted maxillary incisors. 2016. 10.1038/sj.bdj.2018.361 29795486

[2] MacPhee C. The incidence of erupted supernumerary teeth in consecutive series of 4000 school children. Br Dent J. 1935;58:59–60.

[3] Ayers E, Kennedy D, Wiebe C. Clinical recommendations for management of mesiodens and unerupted permanent maxillary central incisors. European archives of paediatric dentistry: official journal of the European Academy of Paediatric Dentistry. 2014;15(6):421–8. Epub 2014/07/06. doi: 10.1007/s40368-014-0132-1 . 24994110 10.1007/s40368-014-0132-1

[4] Leyland L, Batra P, Wong F, Llewelyn R. A retrospective evaluation of the eruption of impacted permanent incisors after extraction of supernumerary teeth. The Journal of clinical pediatric dentistry. 2006;30(3):225–31. Epub 2006/05/11. . 16683671 10.17796/jcpd.30.3.60p6533732v56827

[5] Lygidakis NN, Chatzidimitriou K, Theologie-Lygidakis N, Lygidakis NA. Epsilonvaluation of a treatment protocol for unerupted maxillary central incisors: retrospective clinical study of 46 children. Eur Arch Paediatr Dent. 2015;16(2):153–64. Epub 2014/11/06. doi: 10.1007/s40368-014-0150-z . 25370386 10.1007/s40368-014-0150-z

[6] Betts A, Camilleri GE. A review of 47 cases of unerupted maxillary incisors. Int J Paediatr Dent. 1999;9(4):285–92. Epub 2000/05/18. . 10815587 10.1111/j.1365-263x.1999.00147.x

[7] Bartolo A, Camilleri A, Camilleri S. Unerupted incisors—characteristic features and associated anomalies. Eur J Orthod. 2010;32(3):297–301. Epub 2009/09/12. doi: 10.1093/ejo/cjp094 . 19745002 10.1093/ejo/cjp094

[8] Topkara A, Sari Z. Impacted teeth in a turkish orthodontic patient population: prevalence, distribution and relationship with dental arch characteristics. European journal of paediatric dentistry: official journal of European Academy of Paediatric Dentistry. 2012;13(4):311–6. Epub 2012/12/29. . 23270290

[9] Mitchell L, Bennett TG. Supernumerary teeth causing delayed eruption—a retrospective study. British journal of orthodontics. 1992;19(1):41–6. Epub 1992/02/01. . 1562577 10.1179/bjo.19.1.41

[10] Mason C, Azam N, Holt RD, Rule DC. A retrospective study of unerupted maxillary incisors associated with supernumerary teeth. The British journal of oral & maxillofacial surgery. 2000;38(1):62–5. Epub 2000/04/28. doi: 10.1054/bjom.1999.0210 . 10783451 10.1054/bjom.1999.0210

[11] Jacobs SG. Radiographic localization of unerupted mandibular anterior teeth. American Journal of Orthodontics and Dentofacial Orthopedics. 2000;118(4):432–8. doi: 10.1067/mod.2000.108783 11029740 10.1067/mod.2000.108783

[12] Taylor NG, Jones AG. Are anterior occlusal radiographs indicated to supplement panoramic radiography during an orthodontic assessment? Br Dent J. 1995;179(10):377–81. Epub 1995/11/25. . 8519560 10.1038/sj.bdj.4808931

[13] Jeremias F, Fragelli CM, Mastrantonio SD, Dos Santos-Pinto L, Dos Santos-Pinto A, Pansani CA. Cone-beam computed tomography as a surgical guide to impacted anterior teeth. Dental research journal. 2016;13(1):85–9. Epub 2016/03/11. doi: 10.4103/1735-3327.174723 ; PubMed Central PMCID: PMCPMC4770477. 26962322 10.4103/1735-3327.174723 PMC4770477

[14] Guideline on prescribing dental radiographs for infants, children, adolescents, and persons with special health care needs. Pediatric dentistry. 2012;34(5):189–91. Epub 2012/12/06. . 23211908

[15] Topouzelis N, Tsaousoglou P, Pisoka V, Zouloumis L. Dilaceration of maxillary central incisor: a literature review. Dent Traumatol. 2010;26(5):427–33. Epub 2010/09/14. doi: 10.1111/j.1600-9657.2010.00915.x . 20831640 10.1111/j.1600-9657.2010.00915.x

[16] Gungormus M, Yolcu U, Aras MH, Halicioglu K. Simultaneous occurrence of compound odontoma and arrested root formation as developmental disturbances after maxillofacial trauma: a case report. Medicina oral, patologia oral y cirugia bucal. 2010;15(2):e398–400. Epub 2009/09/22. . 19767697

[17] Shaked I, Peretz B, Ashkenazi M. Development of odontoma-like malformation in the permanent dentition caused by intrusion of primary incisor—a case report. Dental traumatology: official publication of International Association for Dental Traumatology. 2008;24(3):e395–7. Epub 2008/05/21. doi: 10.1111/j.1600-9657.2008.00564.x . 18489475 10.1111/j.1600-9657.2008.00564.x

[18] Flores MT, Malmgren B, Andersson L, Andreasen JO, Bakland LK, Barnett F, et al Guidelines for the management of traumatic dental injuries. III. Primary teeth. Dent Traumatol. 2007;23(4):196–202. Epub 2007/07/20. doi: 10.1111/j.1600-9657.2007.00627.x . 17635351 10.1111/j.1600-9657.2007.00627.x

[19] Kobayashi H, Taguchi Y, Noda T. Eruption disturbances of maxillary permanent central incisors associated with anomalous adjacent permanent lateral incisors. International journal of paediatric dentistry / the British Paedodontic Society [and] the International Association of Dentistry for Children. 1999;9(4):277–84. Epub 2000/05/18. . 10815586 10.1111/j.1365-263x.1999.00146.x

[20] Torres JN, Caracas HC, Bolognese AM, Torres S. Conservative approach for a patient with extreme delay in maxillary lateral incisor development. American journal of orthodontics and dentofacial orthopedics: official publication of the American Association of Orthodontists, its constituent societies, and the American Board of Orthodontics. 2012;141(6):773–82. Epub 2012/05/30. doi: 10.1016/j.ajodo.2010.08.019 . 22640679 10.1016/j.ajodo.2010.08.019

[21] Society. MotCSCotBO. Managing the Developing Occlusion—A guide for dental practitioners: British Orthodontic Society Revised and Updated February 2010.

[22] Pavoni C, Franchi L, Lagana G, Baccetti T, Cozza P. Management of impacted incisors following surgery to remove obstacles to eruption: a prospective clinical trial. Pediatric dentistry. 2013;35(4):364–8. Epub 2013/08/13. . 23930638

[23] Pinho T, Neves M, Alves C. Impacted maxillary central incisor: surgical exposure and orthodontic treatment. American journal of orthodontics and dentofacial orthopedics: official publication of the American Association of Orthodontists, its constituent societies, and the American Board of Orthodontics. 2011;140(2):256–65. Epub 2011/08/02. doi: 10.1016/j.ajodo.2009.11.018 . 21803264 10.1016/j.ajodo.2009.11.018

[24] Nuvvula S, Melkote TH, Mohapatra A, Nirmala SV. Impacted mandibular permanent incisors related to supernumerary teeth: a rare condition. Pediatric dentistry. 2012;34(1):70–3. Epub 2012/02/23. . 22353462

[25] Sant'Anna EF, Marquezan M, Sant'Anna CF. Impacted incisors associated with supernumerary teeth treated with a modified Haas appliance. Am J Orthod Dentofacial Orthop. 2012;142(6):863–71. Epub 2012/12/01. doi: 10.1016/j.ajodo.2011.07.030 . 23195372 10.1016/j.ajodo.2011.07.030

[26] Rohilla M, Namdev R, Dutta S. Dentigerous cyst containing multiple impacted teeth: a rare case report. Journal of the Indian Society of Pedodontics and Preventive Dentistry. 2011;29(3):244–7. Epub 2011/10/12. doi: 10.4103/0970-4388.85834 . 21985882 10.4103/0970-4388.85834

[27] Jena AK. Management of Multiple Impacted Teeth Associated with a Large Dentigerous Cyst in the Maxilla. Journal of dentistry for children (Chicago, Ill). 2015;82(3):157–62. Epub 2016/01/06. . 26731252

[28] Sharma D, Garg S, Singh G, Swami S. Trauma-induced dentigerous cyst involving an inverted impacted mesiodens: case report. Dental traumatology: official publication of International Association for Dental Traumatology. 2010;26(3):289–91. Epub 2010/06/25. doi: 10.1111/j.1600-9657.2010.00882.x . 20572846 10.1111/j.1600-9657.2010.00882.x

[29] Shun Y. Dentigerous cyst associated with an impacted anterior maxillary supernumerary tooth. Journal of dentistry for children (Chicago, Ill). 2008;75(1):104–7. Epub 2008/05/29. . 18505659

[30] Ferrazzano GF, Cantile T, Roberto L, Baldares S, Manzo P, Martina R. An impacted central incisor due to supernumerary teeth: a multidisciplinary approach. European journal of paediatric dentistry: official journal of European Academy of Paediatric Dentistry. 2014;15(2 Suppl):187–90. Epub 2014/08/08. . 25101499

[31] Ferres-Amat E, Maura-Solivellas I, Prats-Armengol J, Ferres-Amat E, Mareque-Bueno J, Ferres-Padro E. Study of the frequency, localisation and morphology of supernumerary teeth in 1960 Spanish non-syndromic paediatric patients. European journal of paediatric dentistry: official journal of European Academy of Paediatric Dentistry. 2015;16(1):19–23. Epub 2015/03/21. . 25793948

[32] Punde PA, Patil NM, Pawar RL. Unusual presentation of 'u-shaped' impacted maxillary central incisor with intranasal root: successful surgical management. Ethiopian journal of health sciences. 2014;24(3):273–6. Epub 2014/09/04. ; PubMed Central PMCID: PMCPMC4141232. 25183935 10.4314/ejhs.v24i3.12 PMC4141232

